# Prepulse inhibition predicts spatial working memory performance in the inbred Roman high- and low-avoidance rats and in genetically heterogeneous NIH-HS rats: relevance for studying pre-attentive and cognitive anomalies in schizophrenia

**DOI:** 10.3389/fnbeh.2015.00213

**Published:** 2015-08-18

**Authors:** Ignasi Oliveras, Cristóbal Río-Álamos, Toni Cañete, Gloria Blázquez, Esther Martínez-Membrives, Osvaldo Giorgi, Maria G. Corda, Adolf Tobeña, Alberto Fernández-Teruel

**Affiliations:** ^1^Medical Psychology Unit, Department of Psychiatry and Forensic Medicine, School of Medicine, Institute of Neurosciences, Autonomous University of BarcelonaBarcelona, Spain; ^2^Section of Pharmaceutical, Pharmacological and Nutraceutical Sciences, Department of Life and Environmental Sciences, University of CagliariCagliari, Italy

**Keywords:** prepulse inhibition, spatial working memory, cognitive deficits, schizophrenia-relevant symptoms, schizophreniform rat model, Roman high-avoidance rats, Roman low-avoidance rats, genetically heterogeneous rats

## Abstract

Animal models of schizophrenia-relevant symptoms are increasingly important for progress in our understanding of the neurobiological basis of the disorder and for discovering novel and more specific treatments. Prepulse inhibition (PPI) and working memory, which are impaired in schizophrenic patients, are among the symptoms/processes modeled in those animal analogs. We have evaluated whether a genetically-selected rat model, the Roman high-avoidance inbred strain (RHA-I), displays PPI deficits as compared with its Roman low-avoidance (RLA-I) counterpart and the genetically heterogeneous NIH-HS rat stock. We have investigated whether PPI deficits predict spatial working memory impairments (in the Morris water maze; MWM) in these three rat types (Experiment 1), as well as in a separate sample of NIH-HS rats stratified according to their extreme (High, Medium, Low) PPI scores (Experiment 2). The results from Experiment 1 show that RHA-I rats display PPI and spatial working memory deficits compared to both RLA-I and NIH-HS rats. Likewise, in Experiment 2, “Low-PPI” NIH-HS rats present significantly impaired working memory with respect to “Medium-PPI” and “High-PPI” NIH-HS subgroups. Further support to these results comes from correlational, factorial, and multiple regression analyses, which reveal that PPI is positively associated with spatial working memory performance. Conversely, cued learning in the MWM was not associated with PPI. Thus, using genetically-selected and genetically heterogeneous rats, the present study shows, for the first time, that PPI is a positive predictor of performance in a spatial working memory task. These results may have translational value for schizophrenia symptom research in humans, as they suggest that either by psychogenetic selection or by focusing on extreme PPI scores from a genetically heterogeneous rat stock, it is possible to detect a useful (perhaps “at risk”) phenotype to study cognitive anomalies linked to schizophrenia.

## Introduction

Schizophrenia symptoms are usually grouped in three categories: positive (hallucinations, delusions, and other thought disorders); negative (anhedonia, avolition, poverty of thought, and content of speech) and cognitive impairment (working memory and attention deterioration). Their complexity, diversity, and bizarreness preclude a full modeling of the entire constellation in animals, just as schizophrenic patients do not manifest every possible symptom. Some of the most commonly used animal models rely on the similarity between the effects that psychotomimetic and psychostimulant drugs trigger in both animals and humans, i.e., those animal analogs mostly reproduce positive (psychotic) symptoms of schizophrenia, while other rodent analogs focus on modeling negative (e.g., impaired social behavior) or cognitive (e.g., impairments of spatial learning, working memory) symptoms of the disorder. In addition, some rat and mouse models may be used to assess sensorimotor gating (pre-attentive) or attention-related processes which are impaired in schizophrenic patients (e.g., reviews by Sawa and Snyder, 2002; Powell and Miyakawa, 2006; Jones et al., 2011; Del Río et al., 2014).

One of these pre-attentive processes is prepulse inhibition (PPI), which refers to the ability of an acoustic stimulus of relatively weak intensity (i.e., prepulse) to diminish the acoustic startle response (ASR) caused by a subsequent acoustic pulse of higher intensity. PPI is an operational measure of the pre-attentive filtering process known as sensorimotor gating, which reflects the neural filtering of redundant or unnecessary stimuli that takes place in complex systems (Freedman et al., 1987; Koch and Schnitzler, 1997; Cromwell et al., 2008; García-Sánchez et al., 2011; Kohl et al., 2013). Since PPI is a cross-species phenomenon, it can be measured in both mammals and humans with the same experimental procedure, thereby providing a very useful paradigm for translational research. PPI is impaired in schizophrenic patients, among other mental disorders, and thus it is widely considered an endophenotype of the disorder (e.g., reviews by Freedman et al., 1987; Koch and Schnitzler, 1997; Cromwell et al., 2008; García-Sánchez et al., 2011; Kohl et al., 2013).

Genetically-based rat models of schizophrenia-related symptoms, derived from selective breeding programs, may have the advantage of symptom stability (within and across generations), and may lead to the identification of clusters of associated/related symptoms. Hence, genetic models represent a useful approach to study the neurobiological and, importantly, genetic mechanisms underlying the symptoms of schizophrenia. Examples of these genetically-based rat models are, for instance, the APO-SUS and APO-UNSUS rat lines (Ellenbroek et al., 1995; van der Elst et al., 2006, the “three hit” Low-PPI rat line (Petrovszki et al., 2013; Kekesi et al., 2015) and the Low-PPI/High-PPI rat lines (Freudenberg et al., 2007; Schwabe et al., 2007), which are rat lines presenting impairments in PPI (APO-SUS, “three hit low-PPI” and Low-PPI) and other schizophrenia-related symptoms, like latent inhibition or some cognitive functions (Ellenbroek et al., 1995; van der Elst et al., 2006; Freudenberg et al., 2007; Schwabe et al., 2007; Petrovszki et al., 2013; Kekesi et al., 2015; see review by Del Río et al., 2014).

The Roman High- (RHA) and Low-avoidance (RLA) rat lines/strains (depending on whether they are outbred –i.e., lines-, or inbred –i.e., strains-), may constitute another of such genetic models. They have been selectively and bidirectionally bred for their rapid (RHA) vs. extremely poor (RLA) ability to acquire the two-way active avoidance task (Bignami, 1965; Broadhurst and Bignami, 1965). The extensive research conducted over the last 40 years (e.g., Zeier et al., 1978; Driscoll and Bättig, 1982; Escorihuela et al., 1995a) has led to the conclusion that anxiety/fear and stress sensitivity are among the most prominent behavioral traits that distinguish the two Roman lines/strains. In fact, compared to their RLA counterparts, RHA rats (both from the outbred line –RHA/Verh- or from the inbred strain –RHA-I-) show a phenotype characterized by: (1) low unconditioned and conditioned anxiety/fear (López-Aumatell et al., 2009a,b,c; Díaz-Morán et al., 2012, 2013c; Martínez-Membrives et al., 2015), (2) a proactive coping style (Steimer and Driscoll, 2003, 2005; Driscoll et al., 2009; Díaz-Morán et al., 2012), (3) decreased sensitivity to reward-loss-induced frustration (e.g., Torres et al., 2005; Rosas et al., 2007; Gomez et al., 2009), (4) lowered activation of the hypothalamus–pituitary–adrenal (HPA) axis in response to stress (Steimer and Driscoll, 2003; Carrasco et al., 2008), (5) enhanced central GABA-A/benzodiazepine complex function (which is known to be critically involved in the regulation of anxiety/frustration; Corda et al., 1997; Bentareha et al., 1998) and (6) increased novelty- and drug-seeking behavior (e.g., Fernández-Teruel et al., 1992, 1997; Escorihuela et al., 1999; Giorgi et al., 2007; Manzo et al., 2014). Most important to assess whether RHA/RLA rats display differential schizophrenia-relevant features, the RHA strain/line displays a poorer performance in several learning/memory tasks (Nil and Bättig, 1981; Driscoll et al., 1995; Escorihuela et al., 1995b; Aguilar et al., 2002) and enhanced impulsive behavior in the 5-CSRTT and DRL-20 operant tasks (Zeier et al., 1978; Moreno et al., 2010; Klein et al., 2014). These profiles suggest that RHA rats may have some value for modeling certain deficits of executive function present in schizophrenia. Compared with the RLA line/strain and/or with standard rat strains, RHA rats display relative deficits in latent inhibition threshold (Fernández-Teruel et al., 2006; and unpublished results from our laboratory), augmented mesocortical dopaminergic response to stress (Giorgi et al., 2003), enhanced locomotor as well as mesolimbic dopaminergic sensitization to repeated (DAergic) psychostimulant administration (Corda et al., 2005; Giorgi et al., 2007; Guitart-Masip et al., 2008), and neurochemical and neuromorphological evidence of decreased hippocampal function (Sallés et al., 2001; Meyza et al., 2009; Garcia-Falgueras et al., 2012). Remarkably, we have recently found that RHA-I rats show a dramatically reduced expression of mGluR2 in prefrontal cortex and hippocampus and increased cortical 5HT2AR expression (Klein et al., 2014). Thus, RHAs display a series of neurobehavioral traits that resemble some schizophrenia—relevant symptoms or associated neural processes.

As said above, schizophrenias usually present (or are associated with) complex clusters of symptoms. Knowing which of them are inter-related or which are orthogonal is important for both, progress in neurobiological research and for the development of novel treatments addressed to particular symptoms or clusters of symptoms. In this context, clinical researchers are studying the relationships among pre-attentive processes, attention, memory, cognition, and executive functions in schizophrenics and healthy human volunteers (Bitsios and Giakoumaki, 2005; Bitsios et al., 2006; Giakoumaki et al., 2008; Csomor et al., 2009). Using this approach it has been shown that PPI may be positively correlated with some cognitive functions, including working memory, in healthy human volunteers (Bitsios and Giakoumaki, 2005; Bitsios et al., 2006; Giakoumaki et al., 2008; Csomor et al., 2009; Singer et al., 2013). In a recent study in mice, Singer et al. have also shown associations between PPI and working memory (Singer et al., 2013).

In the present study we aimed at evaluating possible links between PPI and working memory in the genetically-selected inbred RHA-I and RLA-I rats (supposedly “altered” because of the psychogenetic selection, and therefore representing a parallel of a “clinical” or “at risk” sample), and in the genetically heterogeneous (i.e., outbred) NIH-HS rat stock as a plausible parallel of a normative and healthy human sample. The genetically heterogeneous NIH-HS rat stock (i.e., “National Institutes of Health Genetically Heterogeneous Rat Stock”) was developed by Hansen and Spuhler (1984) through an eight-way cross from eight inbred rat strains and they were bred for more than 50 generations. The NIH-HS rats are a unique tool to study the genetic basis of complex traits due to their broad phenotypic variation and high degree of genetic recombination compared to the usual laboratory rat strains (e.g., Spuhler and Deitrich, 1984; López-Aumatell et al., 2008, 2009a,b, 2011; Johannesson et al., 2009; Vicens-Costa et al., 2011; Díaz-Morán et al., 2012, 2013a,b,c, 2014; Baud et al., 2013, 2014a,b; Estanislau et al., 2013; Palència et al., 2013; Alam et al., 2014; Tsaih et al., 2014). Moreover, NIH-HS rats have been shown to closely resemble RLA-I rats in their coping style and stress sensitivity profiles (e.g., López-Aumatell et al., 2009a; Díaz-Morán et al., 2012, 2013c; Estanislau et al., 2013). Taking into account these characteristics, we used the NIH-HS rats because of their potentially high translational value.

Therefore, the aim of this research was to investigate possible sensory gating-working memory relationships by: (1) characterizing the performance of the three rat strains/stocks (RHA-I, RLA-I, and NIH-HS) for PPI and spatial working memory in the delayed-matching-to-place in the Morris Water Maze (MWM); and (2) evaluating PPI-working memory associations in a sample of heterogeneous NIH-HS rats stratified by their extreme (low or high) PPI levels.

On the basis of the results reviewed above, we hypothesized that (1) RHA-I rats would show PPI and working memory deficits as compared to RLA-I and NIH-HS rats (note that, as said above, RLA-Is and NIH-HS have a very similar behavioral/neuroendocrine profiles in several tests/tasks), and (2) PPI levels would be positively associated with spatial working memory.

## Materials and methods

### Animals

The animals used were males of the inbred Roman High- (RHA-I) and Low-Avoidance (RLA-I) rat strains and the genetic heterogeneous rat stock (NIH-HS, “National Institutes of Health Genetically Heterogeneous Rat Stock”; derived from crossing the MR/N, WN/N, WKY/N, M520/N, F344/N, ACI/N, BN/SsN, and BUF/N strains; Hansen and Spuhler, 1984), from the permanent colonies maintained at our laboratory (Medical Psychology Unit, Dept. Psychiatry, and Forensic Medicine, School of Medicine, Autonomous University of Barcelona) since 1996 (RHA-I, RLA-I) and 2004 (NIH-HS), respectively. They were approximately 4 months old at the beginning of the experiments (weight range 320–420 g), and were housed in same-sexed pairs in standard (50 × 25 × 14 cm) macrolon cages. They were maintained under a 12:12 h light-dark cycle (lights on at 08:00 a.m.), with controlled temperature (22 ± 2°C) and humidity (50–70%) and with free access to food and water.

In Experiment 1 subjects were male rats, from the RHA-I (*n* = 16) and RLA-I (*n* = 19) strains and from the NIH-HS genetically heterogeneous rat stock (*n* = 30), which were submitted to PPI testing and to the working memory tasks (see below). The same RHA-I and RLA-I rats were tested in the cued leaning task, jointly with 17 NIH-HS rats that were randomly selected from the initial sample of 30 animals.

In Experiment 2 subjects were 78 NIH-HS rats which were tested for PPI. From these, 33 NIH-HS rats were randomly selected to be evaluated in the working memory task. From these, 5–6 randomly selected rats from each of the three subgroups formed (see details below) underwent the cued learning task.

Rats in the RHA-I and RLA-I groups came from at least 10 different litters/strain, while NIH-HS rats were from 30 litters in each experiment.

The experiments were performed from 9:00 to 18:00 h. and were approved by the committee of Ethics of the Autonomous University of Barcelona in accordance with the European Communities Council Directive (86/609/EEC) regarding the care and use of animals for experimental procedures.

### Prepulse inhibition of the acoustic startle response

Four sound-attenuated boxes (SR-Lab Startle Response System, San Diego Inst., San Diego, USA) were used. Each box consists of a Plexiglas cylinder situated on the top of a platform with a sensor that detects the strength made by the rat in each trial. Two speakers situated 15 cm from each side of the cylinder deliver the acoustic stimuli and a white noise generator provides the background noise. Each box is constantly lit by a 10 W lamp. The data are transduced by an accelerometer into a voltage which is amplified, digitized, and saved into a computer for further analysis.

The startle session starts with a 5 min habituation period in the startle chambers. Then, 10 “pulse-alone” trials (105 dB, 40 ms) are delivered in order to obtain a basal measure of the ASR (BASELINE 1). After this, each one of the six different types of trials are randomly administered 10 times (60 trials in total):

1. Pulse-alone trials (105 dB 40 ms, BASELINE 2, this was the variable used to calculate the %PPI; see the formula below).
2. Prepulses of 65/70/75/80 dB (20 ms) followed by the startle stimulus (105 dB, 40 ms), with an inter-stimulus interval of 100 ms.
3. No stimulus trials (background noise 55 dB).

At the end, in order to measure the habituation to the startle stimulus, five “pulse-alone” trials were delivered (BASELINE 3).

The interval between trials was 10–20 s with a mean of 15 s. The startle magnitude was recorded during 200 ms after the onset of the pulse.

The degree of PPI (in percentage) is calculated according to the formula:

$$\begin{array}{l} \%PPI=100-\left(\frac{startle\;amplitude\;on\;prepulse\;trials}{startle\;amplitude\;on\;pulse\;trials}\times 100\right) \end{array}$$

### Spatial working memory (delayed matching-to-place task; DMP) and cued learning in the morris water maze

The Morris water maze test was performed in a circular water tank (140 cm in diameter and 30 cm deep) filled with water (24°C) made opaque with white paint.

The animals were tested on 3 consecutive days. They were allowed to swim for 90 s or until they located a platform (diameter 16 cm; height 28 cm) submerged (2 cm) in a fixed position each day. Each rat went through 2 trials per day: a sample/acquisition trial and a retention trial. The two trials were separated by 30 s and the rat was allowed to stay on the platform for 15 s and then spent another 15 s in an individual cage before the second trial started.

Three platform positions were defined: the first day the platform was located in the center of the NW quadrant, the second day it was located at a distance of 15 cm in the S direction and the third day the platform was at the center of the tank. Three starting positions were also defined: S, E, and W, respectively. The starting point and the location of the platform were pseudo-randomly varied each day.

Several room cues were constantly visible from the pool. Escape latencies, path lengths, and swimming speed from each rat and trial were provided by a tracking system (Smart v.2.5.14; PANLAB, Barcelona, Spain) connected to a video camera placed above the pool. Two variables (highly associated to each other) were computed as indexes of spatial working memory: “Mean T1-T2,” distance savings in T2 vs. T1 (i.e., subtraction T1-T2) averaged for the 3 days. “Mean %DP T1-T2,” difference of percentage of distance traveled in the periphery between T1 and T2, averaged for the 3 days.

#### Cued learning

This task consisted of four consecutive trials at 15 min intervals on 2 consecutive days (i.e., 8 trials in total). For this test, the platform protruded 1 cm above the surface of the water and was cued with a small striped (black and white) flag. Black curtains were drawn to minimize the availability of extra-maze cues. There was one platform position (center of the SW quadrant) for the 2 days and 4 starting positions (N, S, E, W). The trials began with the rat facing the wall at the starting point and, if after the maximum allocated time (90 s) the animal had not found the platform, it was gently guided to its position by the experimenter. The parameter used in this task was the distance to reach the platform in each trial. This task is used to see if the animals had any visual, motor, or motivational problems.

### Statistical analysis

Statistical analysis was performed using the “Statistical Package for Social Science” (SPSS, version 17).

Pearson's correlation coefficients were performed among the main variables of both experiments. Multiple linear regression and factorial (direct oblimin; oblique rotation) analyses were applied to data from Experiment 2.

Repeated measures ANOVAs, with the 4 prepulse intensities as a within-subject factor (“3 strains” × “4 prepulse intensities” ANOVA) or with the 3 baseline startle trial blocks as within-subject factor (“3 strains” × “3 baseline startle blocks” ANOVA), were used to evaluate the results from the PPI session.

As differences in navigation speed were observed among the experimental groups (data not shown) in the working memory and cued learning tasks, we have taken the “distance traveled” to reach the platform as the main dependent variable from both experiments. For spatial working memory measures, repeated measures ANOVAs with “T1-T2” (mean distance traveled in the 3 first trials –T1- and three second trials –T2- of each trial pair) as within-subject factor (“3 groups” × “2 trial means” ANOVA) were applied. One-Way ANOVAs were then separately applied to T1 and T2 results (Experiment 1).

Analysis of “distance traveled through the periphery” along the 6 training trials of the working memory task and analysis of performance along the 8 trials of the cued learning task were carried out with the appropriate repeated measures ANOVAs with 6 or 8 trials as within-subject factors (i.e., “3 groups” × “6 trials” or “3 groups” × “8 trials”). *Post-hoc* LSD tests following significant ANOVA effects were applied for comparisons between groups.

## Results

### Experiment 1

Pearson's correlation coefficients between the main variables and for the three groups pooled are shown in Table 1. As expected, there are high within-test (or within-phase) correlations, ranging from *r* = 0.73 to *r* = 0.83 for baseline startle (and habituation) variables, and ranging from *r* = 0.72 to *r* = 0.94 for PPI parameters. There are also moderate correlations between the distance traveled in “T1” and “T2” and in the cued learning task (ranging from *r* = 0.40 to *r* = 0.45), but very low or no correlation between performance in the cued task and the working memory (“Mean T1-T2”) index (*r* = −0.02 and *r* = −0.29). With regard to between-test associations, there are only moderate correlations among %PPI variables and “T2” performance in the working memory task (from *r* = −0.31 to *r* = −0.38).

**Table 1:** **Pearson's correlations among the main variables**.

	**1**	**2**	**3**	**4**	**5**	**6**	**7**	**8**	**9**	**10**	**11**	**12**	**13**	**14**	**15**	**16**	**17**
1-Baseline 1	1																
2-Baseline 2	0.76^**^	1															
3-Baseline 3	0.73^**^	0.83^**^	1														
4-Habituation	0.82^**^	0.43^**^	0.30^*^	1													
5-%PPI65	0.26^*^	0.24	0.18	0.22	1												
6%PPI 70	0.32^**^	0.36^**^	0.27^*^	0.23	0.72^**^	1											
7-%PPI75	0.25^*^	0.27^*^	0.22	0.19	0.87^**^	0.79^**^	1										
8-%PPI80	0.18	0.19	0.11	0.16	0.75^**^	0.79^**^	0.81^**^	1									
9-PPI Mean	0.29	0.30^*^	0.23	0.23	0.92^**^	0.90^**^	0.94^**^	0.89^**^	1								
10-Mean DIST T1	−0.08	−0.05	−0.15	0.02	−0.18	−0.15	−0.11	−0.05	−0.15	1							
11-Mean DIST T2	−0.10	−0.08	−0.15	−0.03	−0.38^**^	−0.31^*^	−0.35^**^	−0.33^**^	−0.38^**^	0.23	1						
12-Mean T1-T2	0.01	0.02	−0.02	0.03	0.13	0.10	0.16	0.20	0.15	0.69^**^	−0.55^**^	1					
13-Mean %DP T1	0.01	0.07	−0.09	0.05	−0.22	−0.10	−0.12	−0.12	−0.16	0.64^**^	0.38^**^	0.27^*^	1				
14-Mean %DP T2	−0.00	0.05	0.00	−0.02	−0.30^*^	−0.19	−0.23	−0.29^*^	−0.27^*^	0.31^*^	0.61^*^	−0.20	0.59^**^	1			
15-Mean %DP T1-T2	0.01	0.02	−0.10	0.07	0.11	0.12	0.13	0.20	0.15	0.32^**^	−0.30^*^	0.50^**^	0.39^**^	−0.52^**^	1		
(^#^)16-Cue day 1	−0.11	0.06	0.05	−0.08	−0.42^**^	−0.32^*^	−0.29^*^	−0.44^**^	−0.41^**^	0.40^**^	0.44^**^	−0.02	0.54^**^	0.63^**^	−0.17	1	
(^#^)17-Cue day 2	0.06	0.33^*^	−0.02	0.16	−0.22	−0.05	−0.12	−0.12	−0.15	0.09	0.45^**^	−0.29^*^	0.45^**^	0.53^**^	−0.15	0.52^**^	1

“Baseline 1,” corresponds to the 10 first pulse-alone trials. “Baseline 2,” corresponds to the 10 pulse-alone trials that are presented pseudorandomly in combination with the prepulse-pulse trials. “Baseline 3,” refers to the last 5 pulse-alone trials. “Habituation” refers to the difference between the mean of the 5 pulse-alone trials presented in the beginning of the session and the mean of the last 5 pulse-alone trials. “%PPI65-%PPI80,” percentage of response inhibition in the prepulse-pulse trials for each prepulse intensity. “PPI Mean,” the global percentage inhibition averaged for the 4 prepulse intensities. “Mean DIST T1” and “Mean DistT2,” mean distance traveled in the three first and the three second trials, respectively. “Mean T1-T2,” distance savings in T2 vs. T1 (i.e., subtraction T1-T2) averaged for the 3 days. “Mean %DP T1” and “Mean %DP T2,” mean of the percentage of the distance traveled in the periphery in the three first (T1) and second (T2) trials, respectively. “Mean %DP T1-T2,” difference of percentage of distance traveled in the periphery between T1 and T2, averaged for the 3 days. “Cue day 1,” mean distance traveled in the 4 trials of cued learning in the first day. “Cue day 2,” mean distance traveled in the 4 trials of cued learning in the second day. Bold letter means significant Pearson's coefficient

* *p ≤ 0.05 and*.

** *p ≤ 0.01. n = 65*.

(#) *n = 41*.

**Figure 1:**
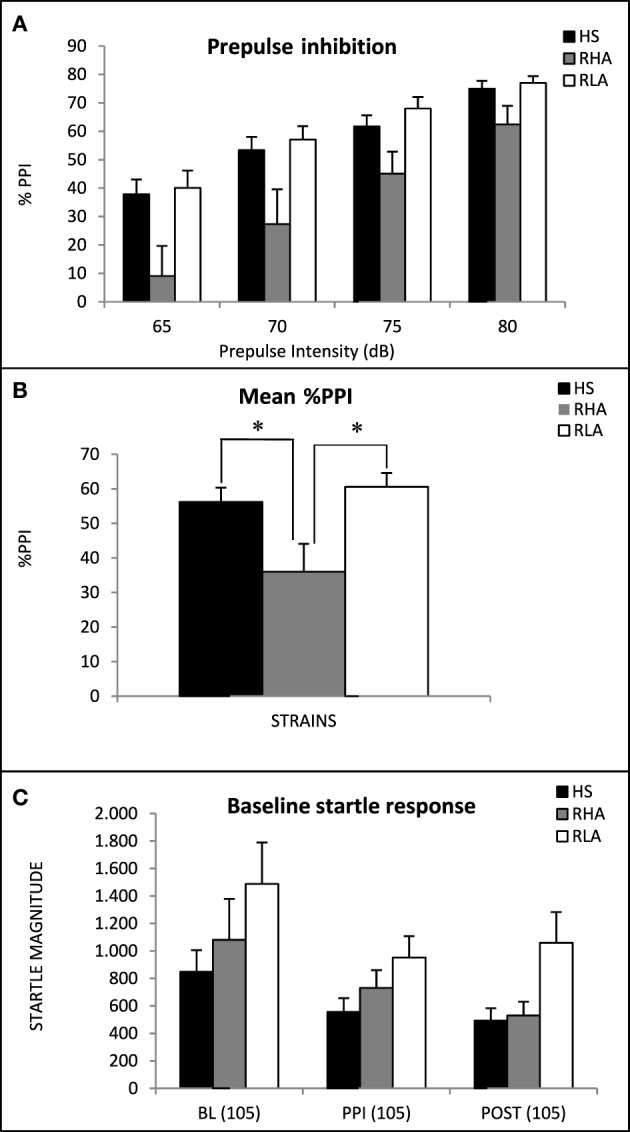
**(A)** Mean prepulse inhibition (± S.E.M.) is shown for the three strains. **(B)** Mean ± S.E.M. of the “Mean %PPI” averaged for the four prepulse intensities. **(C)** Mean ± S.E.M. of the startle response in the three blocks of pulse-alone trials: BL(105), initial pulse-alone 10-trial block; PPI(105), second pulse-alone 10-trial block, pseudorandomly administered in combination with presentation of the prepulse-pulse trials; POST(105), the final pulse-alone 5-trial block. ^*^p < 0.05 between the indicated groups (LSD tests).

The %PPI for each experimental group and prepulse intensity is represented in Figure 1A. The repeated measures ANOVA revealed a significant “strain” effect [*F*_(2, 62)_ = 5.43 *p* < 0.007; Figure 1A]. There was also the expected “prepulse intensity” effect [Huynh-Feldt *F*_(2.56, 166.54)_ = 92.1, *p* < 0.001], as %PPI increased with prepulse intensity (Figure 1A). One-Way ANOVA of the “Mean %PPI” for the 4 prepulse intensities revealed a significant “strain” effect [*F*_(2, 62)_ = 5.43 *p* = 0.007], and the LSD *post-hoc* tests showed that the RHA-I rats display lower mean %PPI than the other two strains (see Figure 1B).

A significant “strain” effect was also observed for baseline startle measures [*F*_(2, 62)_ = 3.35 *p* = 0.041; Figure 1C], which is apparently due to the fact that RLA-I rats display increased baseline startle along the three trial blocks in which the session was divided (in agreement with previous studies; López-Aumatell et al., 2009a,c). There was also a “trial block” effect [Huynh-Feldt *F*_(1.44, 93.5)_ = 16.27 *p* < 0.001], reflecting the habituation of the startle response along the session in the three experimental groups (Figure 1C].

**Figure 2:**
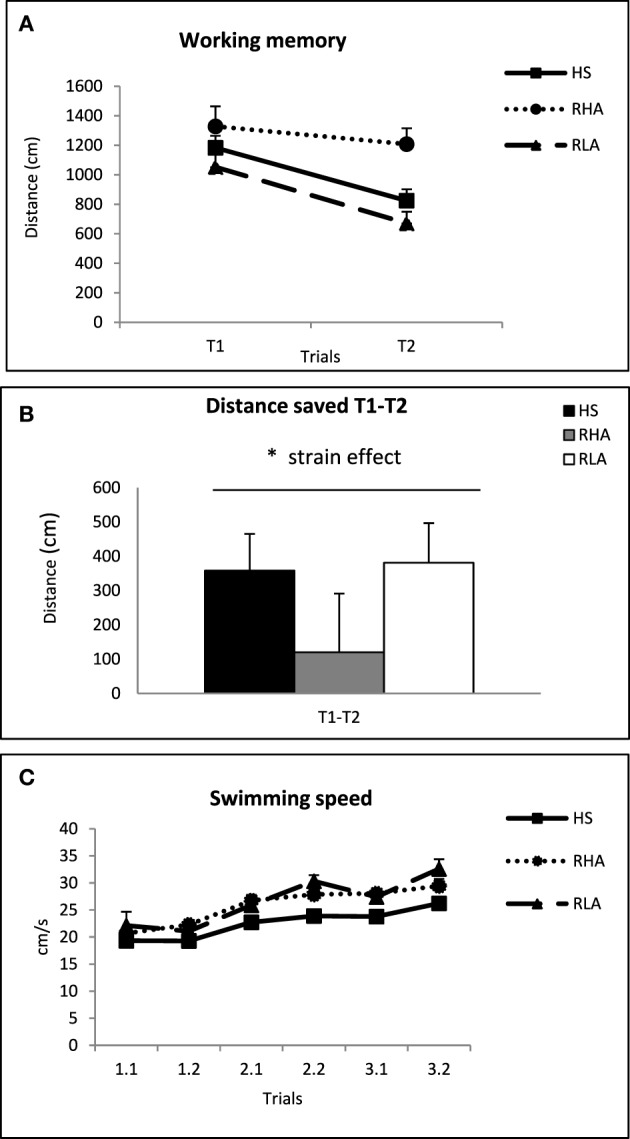
**(A)** Mean ± S.E.M. of the distance (cm) traveled by the rats of the three strains in the first (T1) and second (T2) trials, averaged for the 3 days. **(B)** Mean ± S.E.M. of the three differences (i.e., subtractions T1-T2), corresponding to the 3 days, between the first (T1) and the second trials (T2) of the working memory task. **(C)** Mean ± S.E.M. swimming speed for each trial and strain. ^*^p < 0.05, “Strain” effect (One-Way ANCOVA; see text).

In Figure 2A the average distance traveled in the first trials (T1) and in the second trials (T2) is represented. The repeated measures ANOVA analysis showed a “trial” effect [*F*_(1, 62)_ = 13.9, *p* < 0.001] and a significant “strain” effect [*F*_(2, 62)_ = 5.74 *p* = 0.005], mainly because RHA-I were overall worse than the other two groups (Figure 2A). Moreover, in order to control for the possible influence of “Mean DIST T1” we conducted a one-way analysis of covariance (ANCOVA), taking the mean distance saved between the first and second trials (Mean T1-T2) as the dependent variable and the “distance traveled in the first trials” (Mean DIST T1) as a covariate. This analysis yielded a significant “strain” effect [*F*_(2, 61)_ = 6.39 *p* = 0.003; Figure 2B], which is apparently due to a relative impairment of RHA-I rats in that measure.

We conducted repeated measures ANOVA on swimming speed data for each trial. A significant “strain” effect [*F*_(2, 62)_ = 13.95 *p* < 0.001] was observed, due to the fact that NIH-HS rats swam apparently slower than both RHA-I and RLA-I groups (see Figure 2C).

**Figure 3:**
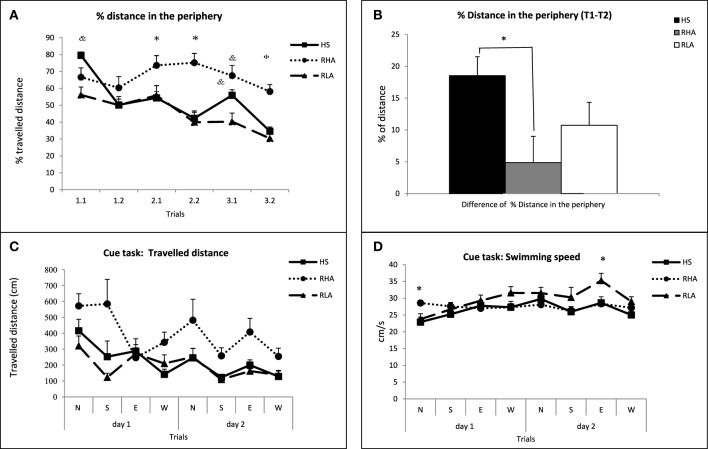
**(A)** Mean ± S.E.M. of the percentage of distance traveled in the periphery in each trial for the three groups. **(B)** Mean ± S.E.M. of the difference of “percentage of distance traveled in the periphery between T1 and T2 (averaged for the 3 days)” (“Mean %DP T1-T2”). **(C)** Mean ± S.E.M. of the distance traveled by the rats in each of the 8 trials of the cued task. **(D)** Mean ± S.E.M. swimming speed in the cued task for each trial. ^*^p < 0.05 vs. the other two strains **(A,D)** or between the groups indicated **(B)**; &, *p* < 0.05 vs. the RLA-I group (LSD tests following the corresponding significant ANOVA effects).

The percentage of distance swam in the periphery, in each trial is shown in Figure 3A. In the repeated measures ANOVA we found a significant “strain” effect [*F*_(2, 62)_ = 12.91 *p* < 0.001]. “Trial” and “trial × strain” effects were also found [*F*_(5, 325)_ = 15.17, *p* < 0.001 and *F*_(10, 325)_ = 5.04, *p* < 0.001, respectively]. Further separate One-Way ANOVAs showed group effects in all trials except one [second trial in the first day, 1.2; *F*_(2, 62)_≥4.11 *p* ≤ 0.021 for the remaining five trials; Figure 3A]. *Post-hoc* LSD tests indicated that RHA-I rats swam longer distances in the periphery than the other two groups (trials 2.1, 2.2, and 3.2; Figure 3A) or than RLA-I rats (trial 3.1; see other LSD differences in Figure 3A). In accordance with the results observed for “Mean T1-T2” (Figures 2A–C), an ANOVA on the “difference of percentage of distance traveled in the periphery between T1 and T2 (averaged for the 3 days)” (“Mean %DP T1-T2”) yielded a significant “strain” effect [*F*_(2, 62)_ = 3.95 *p* < 0.024; Figure 3B], and the LSD *post-hoc* tests revealed that RHA-I rats showed significantly lower values in that variable than NIH-HS rats (Figure 3B). Given that the “Mean %DP T1-T2” variable is highly and positively correlated/associated with “Mean T1-T2” (in both Experiments 1 and 2; see correlations between both variables in Table 1 -*r* = 0.50-, **Table 4** -*r* = 0.69-, and the loadings of both variables −0.79 and 0.81- in the factor analysis from **Table 6B**), the impairment of RHA-I rats in the former variable suggest a relative deficit of RHA-I rats in a working memory-related process.

The repeated measures ANOVA of the distance traveled in the cued task (Figure 3C) revealed significant “strain” [*F*_(2, 38)_ = 11.18 *p* < 0.001] and “trial” [Huynh-Feldt *F*_(4.62, 175.38)_ = 6.44 *p* < 0.001] effects, apparently due to the longer distances traveled by RHA-I rats in some trials. Analysis of the swimming speed of the rats during the cued task (repeated measures ANOVA) indicated a significant “trial × strain” interaction [*F*_(14, 252)_ = 2.01 *p* = 0.018]. Further LSD *post-hoc* tests revealed significant differences between RHA-I rats and the other two strains in the first trial and between the RLA-I and the other two strains in the seventh trial (see LSD tests in Figure 3D).

### Experiment 2

Table 2 shows the mean, S.E.M., and Standard Deviation (S.D.) %PPI of the 78 NIH-HS rats tested in the PPI session.

**Table 2:** **Mean, S.E.M. and Standard Deviation (S.D.) of the 78 NIH-HS rats tested in the PPI**.

	**Mean**	***S.E.M***	***S.D.***
**STARTLE RESPONSE**			
Baseline 1	899.93	114.92	1014.96
Baseline 2	590.66	74.61	658.98
Baseline 3	474.84	58.74	518.76
Habituation	350.74	72.19	637.54
**PPI**			
%PPI65	41.29	3.20	28.24
%PP70	56.34	2.26	19.95
%PPI75	67.71	2.03	17.92
%PPI80	78.50	1.41	12.43
PPI Mean	60.96	2.01	17.78

*Symbols/abbreviations as in Table 1*.

Table 3 shows the mean ± S.E.M of the three sub-groups of NIH-HS rats stratified on the basis of their mean %PPI performance with the 4 prepulse intensities. The 3 sub-groups were formed as follows: HIGH-PPI group (*n* = 9), consisting of rats showing total PPI scores 1 SD above the mean of the whole group (*n* = 78), i.e., PPI scores > 78%; LOW-PPI group (*n* = 14), consisting of rats showing total PPI scores 1 SD below the mean of the whole group, i.e., PPI scores < 42%; MEDIUM-PPI group (*n* = 10), consisting of randomly selected rats with total PPI scores falling within the mean ± 1 SD (see descriptives of the 3 subgroups in Table 3). The results listed in Table 3 show that the 2 groups with extreme values of %PPI also diverge in the magnitude of the baseline startle response.

**Table 3:** **Mean ± S.E.M. of the PPI and startle response variables of the 3 NIH-HS subgroups**.

	**Medium-PPI (n: 10)**	**High-PPI (n: 9)**	**Low-PPI (n: 14)**
Baseline 1	606.24±263.68	1550.98±392.36^*^	571.63±122.74
Baseline 2	256.84±64.03	1194.12±225.94^*^	361.22±88.58
Baseline 3	348.54±117.13	825.42±254.31	381.64±91.00
Habituation	352.66±250.32	807.18±349.05	303.04±112.32
%PPI65	42.88±4.74^a^	74.98±2.97^a^	10.30±4.26^a^
%PP70	62.66±4.39^a^	83.77±1.11^a^	27.14±3.88^a^
%PPI75	68.84±3.69^a^	87.79±0.91^a^	39.91±2.91^a^
%PPI80	79.62±3.82^a^	91.85±1,00^a^	60.88±2.50^a^
PPI Mean	63.50±3.42^a^	84.60±1.11^a^	34.56±1.48^a^

*Symbols/abbreviations as in Table 1*.

* *P < 0.05 vs. the other 2 groups*;

a *p < 0.05 between groups with the same letter (LSD tests following significant One-Way ANOVAs; all [Fs_(2, 30)_ ≥ 4.57 and p ≤ 0.019]*.

The correlation matrix in Table 4 shows a pattern of high within-test correlations which is similar to that observed in Table 1 (Experiment 1). Apart from these expected correlations it is remarkable that the “Mean T1-T2” is positively and significantly correlated with the PPI variables (i.e., %PPI70, %PPI75, %PPI80, and PPI Mean), with correlations ranging from 0.39 to 0.53. So, PPI is correlated with a typical measure of working memory such as the “Mean T1–T2.” Another related correlation, also supporting the main hypothesis of the present study, is the one between the mean distance traveled in the second trial (Mean DIST T2) and the performance in the PPI when the prepulse had an intensity of 75 dB (−0.36). In the case of the other prepulse intensities the correlation with “Mean DIST T2” is not significant but there is a tendency, with Pearson's coefficients ranging from −0.25 to −0.33. Remarkably, the “difference of percentage of distance traveled in the periphery between T1 and T2 averaged for the 3 days” (“Mean % DP T1-T2”), which is highly correlated with the working memory measure “Mean T1-T2” (*r* = 0.69), is also positively correlated with %PPI (i.e., %PPI75, %PPI80 and PPI Mean), with Pearson's coefficients ranging from 0.41 to 0.57. Moreover, correlations between measures of (total) distance (i.e., Mean DIST T1, Mean DIST T2) and “distance traveled in the periphery” (Mean %DP T1, Mean %DP T2) are significant, with coefficients ranging from 0.61 (between Mean DIST T2 and Mean %DP T2) to 0.68 (between Mean DIST T1 and Mean % DP T2; Table 4).

**Table 4:** **Pearson's correlations among the main variables, pooling the three NIH-HS subgroups (*n* = 33)**.

	**1**	**2**	**3**	**4**	**5**	**6**	**7**	**8**	**9**	**10**	**11**	**12**	**13**	**14**	**15**	**16**	**17**
1-Baseline 1	1																
2-Baseline 2	0.83^**^	1															
3-Baseline 3	0.86^**^	0.79^**^	1														
4-Habituation	0.81^**^	0.51^**^	0.46^**^	1													
5-%PPI65	0.45^**^	0.53^**^	0.37^*^	0.33	1												
6-%PPI 70	0.35^*^	0.44^*^	0.23	0.23	0.86^**^	1											
7-%PPI75	0.29	0.41^*^	0.23	0.16	0.83^**^	0.85^**^	1										
8-%PPI80	0.18	0.37^*^	0.14	0.10	0.73^**^	0.78^**^	0.85^**^	1									
9-PPI Mean	0.36^*^	0.48^**^	0.28	0.24	0.94^**^	0.95^**^	0.94^**^	0.88^**^	1								
10-Mean DIST T1	0.21	0.23	0.13	0.23	0.04	0.21	0.36^*^	0.37^*^	0.23	1							
11-Mean DIST T2	0.09	0.01	0.10	0.15	−0.25	−0.32	−0.36^*^	−0.33	−0.33	0.08	1						
12-Mean T1-T2	0.10	0.17	0.03	0.07	0.21	0.39^*^	0.53^**^	0.52^**^	0.42^*^	0.70^**^	−0.65^**^	1					
13-Mean %DP T1	0.36^*^	0.34	0.27	0.29	0.20	0.31	0.37^*^	0.38^*^	0.32	0.68^**^	0.14	0.42^*^	1				
14-Mean %DP T2	0.09	0.02	0.05	0.13	−0.09	−0.08	−0.15	−0.23	−0.13	0.18	0.61^**^	−0.30	0.50^**^	1			
15-Mean %DP T1-T2	0.20	0.26	0.18	0.01	0.26	0.34	0.48^**^	0.57^**^	0.41^*^	0.38^*^	−0.56^**^	0.69^**^	0.28	−0.69^**^	1		
(^#^) 16-Cue Day 1	0.36	0.21	0.32	0.37	0.17	0.13	0.22	−0.01	0.15	0.32	0.18	0.13	0.66^**^	0.66^**^	−0.27	1	
(^#^) 17-Cue Day 2	−0.01	0.32	−0.15	0.01	0.47^*^	0.41	0.41	0.29	0.44	−0.12	0.18	−0.20	0.15	0.19	−0.11	0.13	1

Symbols/abbreviations as in Table 1. Bold letter means significant Pearson's coefficient

* p ≤ 0.05 and

** *p ≤ 0.01*.

(#) *n = 17*.

**Figure 4:**
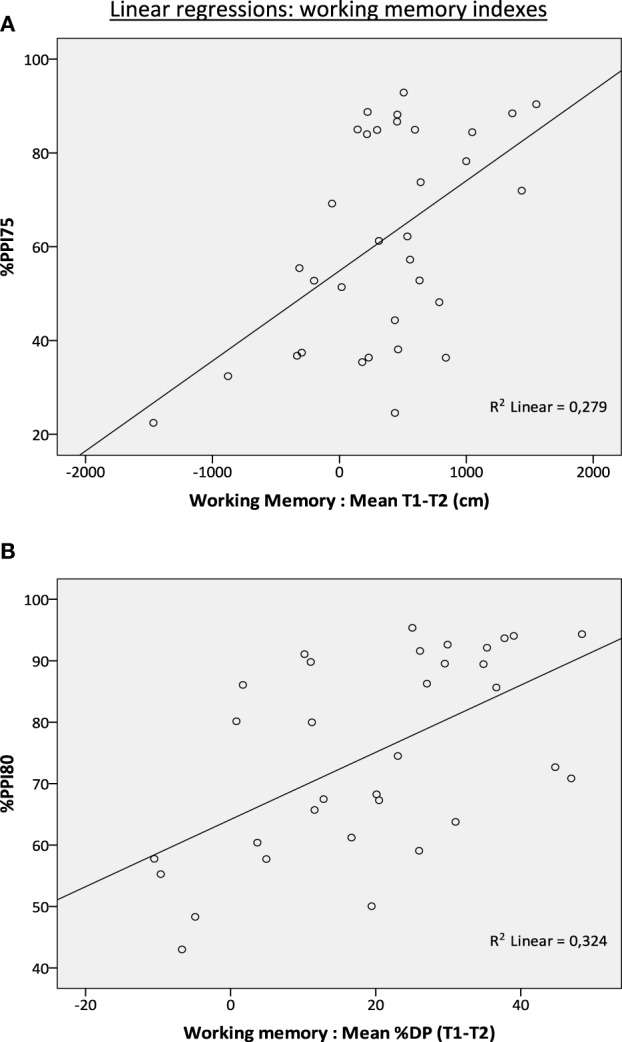
**Linear regressions between %PPI and “working memory” measures (all rats pooled, ***n*** = 33)**. **(A)** Regression between %PPI75 (predictor variable) and “Mean T1-T2” (dependent variable). **(B)** Regression between %PPI80 (predictor variable) and “Mean %DP T1-T2” (dependent variable).

Forward stepwise multiple regression, with PPI variables as predictors and “Mean T1-T2” as the dependent variable revealed 2 significant models, with the first model showing that the variable %PPI75 is a significant predictor of “Mean T1-T2” (see Figure 4A, Table 5). %PPI75 correlated significantly with “Mean T1-T2,” explaining 28% of its variability (*p* = 0.002; Table 5). In addition, a second significant regression model, with %PPI75 and %PPI65 as predictors (correlation of *r* = 0.66 with “Mean T1-T2”) accounted for 44% of the variability (*p* < 0.001; Table 5). We also conducted the multiple regression analysis for the “Mean %DP T1-T2” and we observed that %PPI80 predicted “Mean %DP T1-T2” (Figure 4B; Table 5).

**Table 5:** **Forward stepwise multiple regression performed with “Mean T1-T2” and “Mean %DP T1-T2” as the dependent variables, and including all the variables recorded in the PPI session (i.e., Baseline 1–3, Habituation and %PPI65–%PPI80) as predictors in the model**.

**Dependent variable**	**Method**	**Model**	**Predictor variables**	***R***	***R*^2^**	***p***
Mean T1-T2	Forward Stepwise	1	%PPI75	0.53	0.28	0.002
Mean T1-T2	Forward Stepwise	2	%PPI75; %PPI65	0.66	0.44	< 0.001
Mean %DP T1-T2	Forward stepwise	1	%PPI80	0.57	0.32	0.001

*Mean T1-T2 is predicted by the PPI variables %PPI75 and %PPI65. Mean %DP T1-T2 is predicted by %PPI80. The three NIH-HS subgroups (n = 33) are pooled for analysis*.

Obliquely-rotated factor analyses (direct oblimin) were performed with the 14 main variables of the two tests/tasks (Table 6(A)). The first result we obtained was a 4-factor structure (Table 6(A)). In the first factor we can see an association between PPI and working memory, with loadings of PPI variables ranging from 0.89 to 0.95 and with 0.43 and 0.41 loadings for “Mean T1-T2” and “Mean %DP T1-T2,” respectively. The second factor is based on the startle response magnitude, particularly baseline startle, with loadings from 0.78 to 0.99. The third and fourth factors are related to the DMP task (loadings ranging from 0.41 to 0.91), with the main difference between the two factors being that in the third the distances in the first trials (Mean DIST T1 and Mean %DP T1) have the highest loadings, while in the fourth factor the distances in the second trials (Mean DIST T2 and Mean %DP T2) are the most relevant. Another important difference between these two factors is that “Mean T1-T2” has a higher loading in the third factor (0.74) while “Mean %DP T1-T2” has a -0.82 loading in the fourth factor. So, in this analysis we found that, with the exception of the first factor, the other three are predominantly task-related (or phase-related) factors (Table 6(A)).

**Table 6:** **Loadings ≥ 0.40 are shown**.

**(A)**	**Factor 1**	**Factor 2**	**Factor 3**	**Factor 4**	**(B)**	**Factor 1**	**Factor 2**
Baseline 1	–	0.99	–	–	Baseline 1	–	0.91
Baseline 2	0.47	0.87	–	–	Baseline 2	–	0.84
Baseline 3	–	0.89	–	–	Baseline 3	–	0.78
Habituation	–	0.78	–	–	Habituation	–	0.73
%PPI65	0.92	0.45	–	–	%PPI65	0.60	0.59
%PPI70	0.95	–	–	–	%PPI70	0.71	0.52
%PPI75	0.94	–	–	–	%PPI75	0.81	0.48
%PPI80	0.89	–	0.41	–	%PPI80	0.83	–
Mean DIST T1	–	–	0.94	–	Mean DIST T1	–	0.41
Mean DIST T2	–	–	–	0.84	Mean DIST T2	−0.72	–
Mean T1-T2	0.43	–	0.74	−0.64	Mean T1-T2	0.79	–
Mean %DP T1	–	–	0.85	–	Mean %DP T1	–	0.60
Mean %DP T2	–	–	–	0.91	Mean %DP T2	−0.55	–
Mean %DP T1-T2	0.41	–	0.44	−0.82	Mean %DP T1-T2	0.81	–
% of variance (cumulative)	39.40	61.01	74.52	85.50	% of variance (cumulative)	39.40	61.01
Correlations	1				Correlation	0.17	
	0.31	1			N =	33	
	0.27	0.18	1				
	−0.27	0.07	−0.07	1			
N =	33						

*(A) Oblique four-factor solution (direct oblimin) with the main behavioral variables and correlations between factors. Only factors with eigenvalues greater than 1 are considered. (B) Two-factor solution and correlation between factors, showing that both factors are almost orthogonal/independent. Symbols/abbreviations as in Table 1. n = 33 (the three NIH-HS subgroups are pooled for the analyses)*.

In order to obtain a reduced number of theoretically meaningful factors the same factor analysis was reduced to a 2-factor solution (see Table 6(B)). In the first factor the PPI variables and the DMP task variables have the highest loadings, with 0.60–0.83 for %PPI65–%PPI80 and, remarkably, with 0.79 for “Mean T1-T2” and 0.81 for “Mean %DP T1-T2” (Table 6(B)). The second factor is essentially composed by the baseline startle variables (0.73–0.91) plus moderate loadings of %PPI65–%PPI75 variables (0.48–0.59). Thus, this factor analysis clearly links PPI and the working memory task in the first factor, while the second factor is mainly related to baseline startle response.

**Figure 5:**
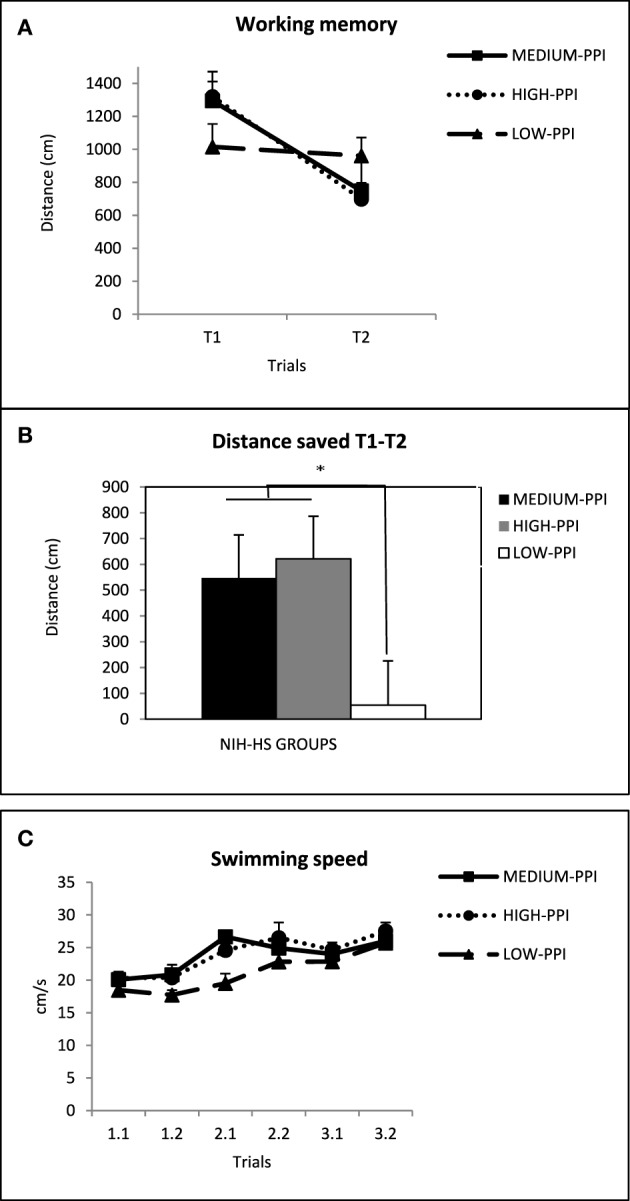
**(A)** Mean ± S.E.M. of the distance (cm) traveled by the three subgroups of NIH-HS rats (selected for their extreme performance in the PPI test) in the first (T1) and second trials (T2), averaged for the 3 days (see text for the criteria followed to build these 3 subgroups). **(B)** Mean ± S.E.M. of the three differences (i.e., “Mean T1-T2”), corresponding to the 3 days, between the first (T1) and second (T2) trials of the working memory task. **(C)** Mean ± S.E.M. swimming speed of the NIH-HS sub-groups for each trial. ^*^p < 0.05 between the indicated groups (LSD test following significant One-Way ANOVA).

In Figure 5A we show the average distance traveled in the first (T1) and second (T2) trials by the three groups of NIH-HS rats. The repeated measures ANOVA showed that there is a significant interaction between “trial type” (T1 or T2) and NIH-HS rat “sub-group” [*F*_(2, 30)_ = 3.44 *p* = 0.045]. This interaction is important because it shows that the “LOW-PPI” group had a very small saving between T1 and in T2, while “HIGH-PPI” and “MEDIUM-PPI” groups show a clear cut decrease in the distance traveled between the two trials (Figure 5A). Further One-Way ANOVAs for each trial type revealed no significant differences between sub-groups. In Figure 5B the differences between T1 and T2 are shown for the three groups. One-Way ANOVA revealed significant differences between the three groups [*F*_(2, 30)_ = 3.44 *p* = 0.045], and *post-hoc* LSD tests evidenced that these differences were between the “LOW-PPI” group and the other two groups (*p* < 0.05; Figure 5B), indicating that the “LOW-PPI” sub-group displays a poorer working memory performance. Analysis of the swimming speed with a repeated measures ANOVA revealed a significant “sub-group” effect [*F*_(2, 30)_ = 7.77 *p* = 0.002], which is due to the fact that the LOW-PPI group was apparently slower than the other two sub-groups in some trials (Figure 5C).

**Figure 6:**
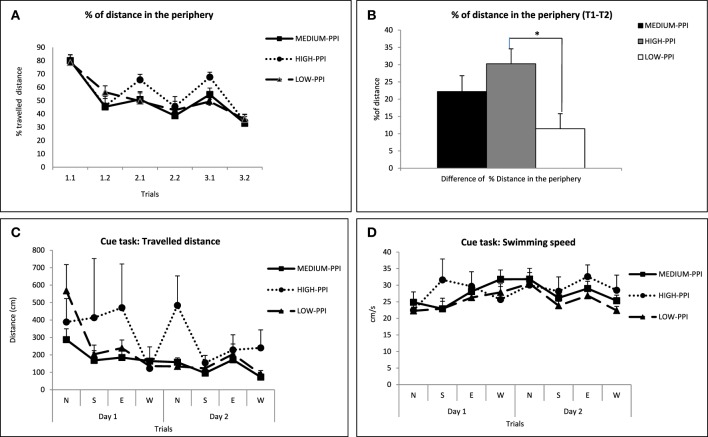
**(A)** Mean ± S.E.M. of the percentage of distance traveled in the periphery in each trial for the 3 groups of NIH-HS rats. **(B)** Mean ± S.E.M. of the “difference of percentage of distance traveled in the periphery between T1 and T2 (averaged for the 3 days)” (i.e., “Mean %DP T1-T2”). **(C)** Mean ± S.E.M. of the distance (cm) traveled by the rats of the three groups in each of the 8 trials of the cued task. **(D)** Mean ± S.E.M. swimming speed of the NIH-HS sub-groups for each trial. ^*^p < 0.05 between the groups indicated (LSD test following significant One-Way ANOVA).

The repeated measures ANOVA for the percentage of distance traveled in the periphery by the three NIH-HS sub-groups revealed no significant group effect [*F*_(2, 30)_ = 0.70 *p* = 0.50] and a significant trial effect [*F*_(5, 150)_ = 28.6 *p* < 0.001] (Figure 6A). On the other hand, One-Way ANOVA of the “Mean %DP T1-T2” showed a significant “sub-group” effect [*F*_(2, 30)_ = 4.51 *p* < 0.019], and the LSD *post-hoc* tests revealed that the differences were between LOW-PPI and HIGH-PPI groups, with the former traveling longer distances in the periphery (Figure 6B).

In Figure 6C we show the distance traveled in the 8 trials of the cued task. The results of the repeated measures ANOVA showed a significant group effect [*F*_(2, 14)_ = 4.37 *p* = 0.033] and a trial effect [Huynh-Feldt *F*_(3.62, 50.78)_ = 2.88 *p* = 0.036; Figure 6C], which is apparently due to HIGH-PPI group traveling longer distances in some trials (see Figure 6C). Swimming speed along the 8 trials was analyzed with a repeated measures ANOVA, that only revealed a “trial” effect [*F*_(7, 98)_ = 3.96 *p* = 0.001; Figure 6D].

## Discussion

Experiment 1 represents the first joint characterization of PPI and spatial working memory in RHA-I/RLA-I and NIH-HS rats. In keeping with the hypothesis that RHA-I rats could be a tool to discern cognitive peculiarities linked to some schizophrenia-related symptoms, we have found that this rat strain displays deficits in PPI and in spatial working memory compared with RLA-I and NIH-HS rats. In addition, it is also shown for the first time that the genetically heterogeneous NIH-HS rat stock displays relatively high PPI levels as well as efficient spatial working memory, as their values in both processes/measures are similar to those shown by RLA-I rats (see Experiment 1). The results from Experiment 1 and Experiment 2 also point out an apparent positive association between PPI and working memory, as the strains (i.e., RLA-I, NIH-HS; Experiment 1) or sub-groups (i.e., the HIGH-PPI and MEDIUM-PPI sub-groups of NIH-HS rats; Experiment 2) displaying the highest PPI levels also present the best working memory performance.

RLA rats from the Swiss outbred line have already been shown to outperform their RHA counterparts in spatial learning/memory tasks (e.g., Driscoll et al., 1995; Escorihuela et al., 1995b; Aguilar et al., 2002). Accordingly, we recently found that inbred RHA-I rats performed worse than RLA-I animals in a spatial place task (i.e., reference memory) in the Morris Water maze (Martínez-Membrives et al., 2015). But the present is the first time in which (spatial) working memory has been assessed in the inbred Roman rat strains, showing that the RHA-I strain displays impaired memory ability in this task as compared with both RLA-I and NIH-HS rats. Remarkably, as we have reported for anxiety/stress-related behavioral/neuroendocrine traits (e.g., López-Aumatell et al., 2009a,b; Díaz-Morán et al., 2012, 2013c; Estanislau et al., 2013), PPI and working memory performance of the heterogeneous NIH-HS rat stock very closely resemble the profile of RLA-I rats in these tests/tasks. These similar profiles, in both a pre-attentive process (PPI) and spatial working memory, jointly with their comparable performance in cued learning, in spatial place learning and in two-way avoidance acquisition (e.g., López-Aumatell et al., 2009a,b; Díaz-Morán et al., 2012, 2013c; Estanislau et al., 2013; Martínez-Membrives et al., 2015), suggest that inbred RLA-I and heterogeneous NIH-HS rats may also present similarities in some other cognitive traits or profiles. Further studies aimed at comparing the performance of RLA-I/RHA-I and NIH-HS rats in other cognitive/executive tasks are therefore warranted.

The RHA-I rat strain also showed a poorer performance in the cue learning task, a test that is commonly used to rule out possible sensorial, motivational, or motor deficits. This result was unexpected, because we have recently found that RHA-I and RLA-I rats present identical performance in a cue task administered after a place task in the MWM (Martínez-Membrives et al., 2015). Apart from that recent work, several studies performed at our laboratory and others allow us to rule out the possibility of any visual, motivational, or motor deficit in RHA-I rats while, on the contrary, it is well-known that they are characterized by: (i) enhanced exploratory drive (motivation) and a novelty seeker profile, as observed in many different novelty situations (e.g., reviews by Fernández-Teruel et al., 1992; Escorihuela et al., 1999; Driscoll et al., 2009); (ii) augmented impulsivity and tendency to stereotyped behavior (Zeier et al., 1978; Moreno et al., 2010; Klein et al., 2014) and (iii) enhanced perseverative responses and/or lack of behavioral flexibility (e.g., Zeier et al., 1978; Nil and Bättig, 1981; Escorihuela et al., 1995b; Moreno et al., 2010). Finally, the “strain × trial” effect (and the absence of “strain” effects) on navigation speed in the cued task (Experiment 1) do not suggest any motivational deficit which could account for the performance results observed. Thus, although it is not the only possible interpretation (see below), the impairment of RHA-I rats in the cue task might be partly due to the fact that they perseverate in (spatial or/and non-spatial) swimming strategies acquired during the working memory task.

In that context, it is worth noting that in both the working memory and cued tasks RHA-I rats display increased circular navigation along the periphery of the pool. This may suggest enhanced thigmotaxis in that rat strain. Nevertheless, we know, from a number of studies using open field-like tests (e.g., open field, activity cages, hole-board), that RHA-I rats spend the same time close to the walls (i.e., in the periphery) and travel the same percentage of distance along the periphery as RLA-I rats (e.g., Estanislau et al., 2013, and unpublished results from our laboratory). Hence, thigmotactic-like behavior is neither an inherent nor a general trait in RHA-I rats but, still, they display an excessive amount of peripheral navigation in the present MWM tasks. Thus, there is the possibility that a working memory deficit in the RHA-I rats may not be the only interpretation of the present results, and that a non-optimal navigation/orientation strategy (which would be in agreement with RHA vs. RLA results in other aquatic spatial tasks; Nil and Bättig, 1981) could have a role in the increased total distance and “% distance in the periphery” traveled by the RHA-I strain.

The results of Experiment 1 also highlight that the RHA-I strain fulfills some criteria for being considered as a convenient model for studying some schizophrenia-relevant symptoms, since it displays clear PPI and (possibly) working memory deficits along with a relative deficit in latent inhibition (Fernández-Teruel et al., 2006), an impaired performance in the five-choice serial reaction time task (5-CSRTT; which reflects executive function and sustained attention; Moreno et al., 2010; Klein et al., 2014) and an increased sensitivity to acutely administered DA receptor agonists as well as to psychostimulant (DA agonist)-induced sensitization (e.g., Corda et al., 2005; Giménez-Llort et al., 2005; Giorgi et al., 2007; Guitart-Masip et al., 2008; Del Río et al., 2014).

Experiment 2 was devoted to further investigate such a PPI-working memory association by evaluating whether PPI levels would statistically predict memory performance in a sample of the most genetically heterogeneous laboratory rat in existence, i.e., the NIH-HS rats (e.g., Baud et al., 2013, 2014a,b; Johannesson et al., 2009). Thus, the expected advantage of addressing that issue in NIH-HS rats would be that their enhanced genetic variability could make results more generalizable than those obtained using typical laboratory rodent strains. The results of Experiment 2 clearly support a positive relationship between PPI and working memory, as shown by the memory performance of HIGH-PPI as compared with MEDIUM-PPI and LOW-PPI rats, as well as by the positive associations between PPI (at different prepulse intensities) and the working memory measures revealed by correlational, factorial, and regression analyses (see Tables 4–6 and Figure 4). In fact, Pearson's correlations between working memory (“Mean T1-T2” index) and %PPI70, %PPI75, %PPI80, and mean %PPI range from *r* = 0.39 to *r* = 0.53 (Table 4). Likewise, the stepwise multiple regression analysis supports this correlational pattern, as the %PPI75 and %PPI65 are significant predictors of both working memory measures, “Mean T1-T2” and “Mean %DP T1-T2” (see Table 5 and Figures 4A,B). Further supporting these results, the factor analysis (oblimin direct; unforced rotation) shows an initial four-fold solution in which, (1) the first factor combines high loadings of %PPI variables (0.89–0.95) with moderate loadings of the working memory variables (i.e., “Mean T1-T2,” loading 0.43; “Mean %DP T1-T2,” loading 0.41) (Table 6(A)); (2) a second factor related to the startle response variables and (3) the last two factors related to the distance variables measured in the MWM. Likewise, when forcing this factor analysis to a two-fold solution (direct oblimin), a very similar first factor is observed, with high loadings of %PPI variables and working memory indexes (“Mean T1-T2”and “Mean %DP T1-T2”), and also high loading of distance traveled in the second trials (“Mean DIST T2” and “Mean %DP T2”). The second and almost orthogonal factor (as the between-factor correlation is low) is mainly grouping loadings of baseline startle, habituation, and moderate loadings in the PPI variables and the distance traveled in the first trials in the MWM (Table 6(B)).

Thus, correlational, regression, and factor analyses confirm that: (1) the differences of % distance traveled in the periphery between T1 and T2 (“Mean %DP T1-T2”) are strongly associated to “Mean T1-T2,” so both of them are working memory indexes; and (2) both working memory variables are positively predicted by %PPI.

As concerns to animal research, to the best of our knowledge the PPI-working memory association has only been addressed in one study in mice and no study has evaluated it in untreated/undisturbed genetically heterogeneous rats. Singer et al. (2013) reported, using a cohort of 23 male C57BL/6 mice, that PPI levels positively predicted (with *r* = 0.50) spatial working memory in the Morris Water maze (delayed matching-to-place task). The authors showed that such an effect was only present when taking as predictor the %PPI levels at the lowest pre-pulse intensity (i.e., 69 dB). Our present results confirm and extend the previous study in mice to different rat types and statistical approaches, as we also found a positive association between %PPI and spatial working memory by comparing the RHA-I, RLA-I, and NIH-HS strains/stocks and by studying PPI-working memory associations in NIH-HS rats.

Remarkably, Experiment 2 shows that cue learning is neither associated with %PPI nor with working memory performance in NIH-HS rats (see Table 4 and Figure 6B). Finally, the extreme LOW-PPI rats from such stock presented a pattern of results both across the different intensities of the prepulse inhibition study and on working memory measures at the DMP test, which paralleled those of the Roman High-Avoidance rats in Experiment 1. This suggests that either by psychogenetic selection or by focusing on extremes from a heterogeneous rats stock, it is possible to detect a useful (perhaps “at risk”) phenotype to study cognitive peculiarities linked to some schizophrenia anomalies.

It has been proposed that PPI may be associated to, and modulated by, higher cognitive processes. This contention is supported by the frequent co-existence of PPI deficits and cognitive impairments in clinical samples, including schizophrenic patients, as well as by some studies in healthy volunteers which have shown that PPI is positively correlated with performance in several cognitive tasks (e.g., Bitsios and Giakoumaki, 2005; Hagan and Jones, 2005; Bitsios et al., 2006; Giakoumaki et al., 2006; Csomor et al., 2009; for reviews see Young et al., 2009; Singer et al., 2013). In particular, in human volunteers, PPI has been found to be positively associated to proper searching strategies in the CANTAB (“*Cambridge Neuropsychological Test Automated Battery”)* spatial working memory task, Csomor et al., 2009). In spite of these positive results, the possibility that PPI predicts cognitive function in humans remains to be established (Young et al., 2009; Singer et al., 2013), given the small number of studies that have addressed that issue.

Koch and Schnitzler (1997) have proposed that the essential circuit underlying PPI involves the midbrain inferior colliculus, the superior colliculus, the pedunculopontine tegmental nucleus, and the caudal pontine reticular nucleus, which regulates the activity of motor neurons and the motor response (Koch and Schnitzler, 1997; Kohl et al., 2013). Importantly, however, cortical, and limbic areas, such as the orbitofrontal cortex, anterior cingulate, medial prefrontal cortex, nucleus accumbens, basolateral amygdala, and the hippocampus are known to modulate PPI or to affect its regulation in different ways, as reflected by disruption of PPI following manipulations of these structures (for review see Kohl et al., 2013). Spatial working memory, as assessed in the DMP task, is known to be hippocampus dependent (Whishaw, 1985; Morris et al., 1986a,b; Wible, 2013). Thus, the finding that PPI and spatial working memory can be modulated by hippocampal function, albeit to a different extent or in different ways, together with the important role attributed to the hippocampus in other schizophrenia-relevant cognitive processes and schizophrenic symptoms (e.g., Gray et al., 1991; Sawa and Snyder, 2002; Wible, 2013), suggests that the hippocampus represents a prime candidate structure to investigate neurobiological processes underlying particular symptom clusters. In this connection, it is remarkable that the RLA-I rat strain has a more functional hippocampus and higher hippocampal neuronal density than the RHA-I strain (Meyza et al., 2009; Garcia-Falgueras et al., 2012), which may underlie the better performance of RLA-I rats in both PPI and spatial working memory.

In summary, the present study demonstrates a consistent and positive PPI-working memory association using three different strategies: (1) comparing three strains/stocks of rats which show differential PPI levels (Experiment 1); (2) evaluating working memory in subsamples of NIH-HS rats displaying extreme scores in PPI; and (3) performing correlational, regression, and factor analysis of PPI and working memory assessed in a sample of genetically heterogeneous NIH-HS rats. The results of the present study, together with those from Singer et al. (2013) in mice, support the idea that PPI and working memory are positively associated in untreated animals, thus paving the path for the study of possible common neurobiological mechanisms of pre-attentive (sensorimotor gating) and higher cognitive processes in rodents that can illuminate routes for abnormal functioning in schizophrenias.

### Conflict of interest statement

The authors declare that the research was conducted in the absence of any commercial or financial relationships that could be construed as a potential conflict of interest.
